# MicroRNA-31 suppresses the self-renewal capability of α2δ1^+^ liver tumor-initiating cells by targeting *ISL1*

**DOI:** 10.18632/oncotarget.21140

**Published:** 2017-09-21

**Authors:** Yuan Zhang, Wei Zhao, Haibo Han, Sheng Li, Dongji Chen, Zhiqian Zhang

**Affiliations:** ^1^ Department of Cell Biology, Key Laboratory of Carcinogenesis and Translational Research (Ministry of Education/Beijing), Peking University Cancer Hospital and Institute, Beijing 100142, China

**Keywords:** MicroRNA, hepatocellular carcinoma, tumor-initiating cell, miR-31, ISL1

## Abstract

Accumulating evidence demonstrates that miRNAs, a class of small non-coding RNAs, are involved in the regulation of tumor-initiating cells (TICs) which are considered to be the origin of cancer development according to the cancer stem cell hypothesis. We have previously identified that miR-31 may play suppressive roles in α2δ1^+^ hepatocellular carcinoma (HCC) TICs. Here, we confirm that the expression of miR-31 is significantly downregulated in α2δ1^+^ HCC TICs. Overexpression of miR-31 in α2δ1^+^ HCC TICs results in significant suppression of the self-renewal and tumorigenicity abilities of these cells. Conversely, knockdown the expression of miR-31 in PLC/PRF/5 cells is able to reprogram them into TICs with stem cell-like properties. Furthermore, the expression of ISL LIM Homeobox 1(ISL1), a transcription factor involved in recognition of undifferentiated cardiac progenitors, is negatively regulated by miR-31, and the luciferase reporters’ activities with the 3′-UTRs of *ISL1* are inhibited significantly by miR-31. Collectively, our results suggest that miR-31 can negatively regulate the self-renewal ability of α2δ1^+^ liver TICs via silencing *ISL1*.

## INTRODUCTION

Hepatocellular carcinoma (HCC) is one of the most aggressive solid tumors associated with poor prognosis, mainly due to its high frequency of recurrence and metastasis [1]. Recent studies indicate that the existing of a rare subset of cancer cells, often operationally called as tumor-initiating cells (TIC) or cancer stem cells, is responsible for sustaining tumor growth and recurrence of many kinds of cancer types including HCC [2–5]. HCC TICs have been isolated and characterized using a variety of stem cell surface markers, such as CD13, CD24, ɑ2δ1(isoform5), EpCAM and CD133 [6–10]. They behave like stem cells in that they are capable of self-renewal and can form heterogeneous tumors *in vivo*, but the regulatory mechanisms are not identical from normal stem cells. But the molecular mechanisms involved in the regulation of HCC TICs is poorly understood.

MiRNAs are an abundant class of small, non-coding RNAs with about 22 nucleotides in length. The predominant function of miRNAs is to negatively regulate gene expression through base pairing with the 3′-untranslated regions (UTRs) of target messenger RNAs (mRNAs) [11]. By acting as tumor promoters or suppressors, a number of miRNAs, such as miR-181a, miR-10b, let-7c, and miR-200b have been found to be involved in multi-aspects of HCC development, such as regulating the acquisition and subsequent maintenance of TIC properties and the progression of cancers [12–14]. In fact, accumulating evidence demonstrates that miRNAs are not only amenable therapeutic targets but also promising prognostic biomarkers [15, 16].

The mircoRNA-31 (miR-31) has been frequently observed to be aberrantly expressed in various human cancers including HCC [17–21]. However, its role in cancer development is controversial in that it acts as an oncogene in some cases, whereas a tumor suppressor role is also appreciated [18, 20]. On the basis of a pair of HCC cell lines Hep-11 and Hep-12, which were established from a single patient's primary and recurrent HCC tissues and represent non-tumorigenic and TIC-enriched cell populations [22], respectively, we identified that miR-31 was remarkably downregulated in the TIC-enriched HCC Hep-12 cell line, and was among those miRNAs that might suppress the properties of α2δ1^+^ HCC TICs, as revealed by combining genome-wide miRNA profiling and soft-agar functional screening assay [14]. Here, we confirm that miR-31 is significantly downregulated in α2δ1^+^ HCC TICs, and serves as a suppressor of α2δ1^+^ HCC TICs via targeting ISL LIM Homeobox 1 (*ISL1*), a transcriptional factor involved in recognition of undifferentiated cardiac progenitors [23, 24].

## RESULTS

### Ectopic expression of miR-31 suppresses the properties of ɑ2δ1^+^ HCC TICs

**Figure 1:**
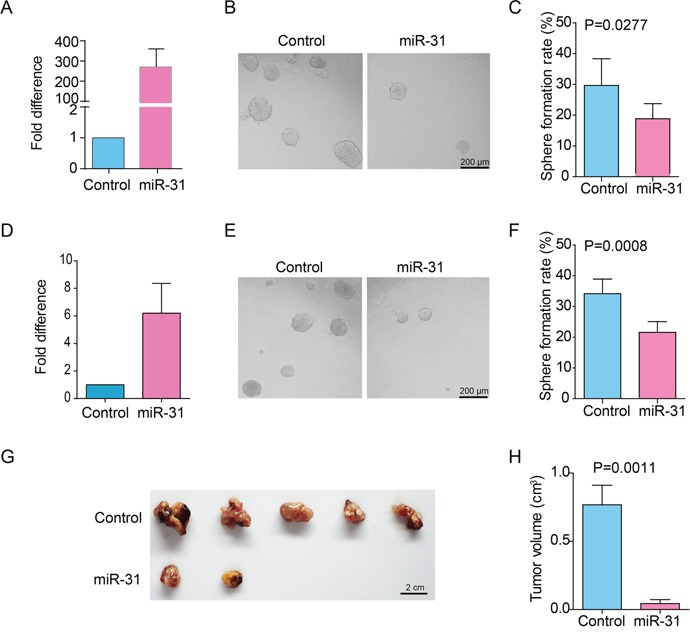
The effects of miR-31 overexpression on the properties of ɑ2δ1+ HCC TICs **(A)** qRT-PCR analysis of the expression of miR-31 in the TIC-enriched Hep-12 cells which were infected with pri-miR-31 or control lentivirus. Data presented as fold change of the cells infected with pri-miR-31 lentivirus over control cells, which was defined as 1 (calibrator). Error bars indicate S.D. **(B)** Representative photographs demonstrating the spheroids formed by Hep-12 cells infected with pri-miR-31 or control lentivirus. **(C)** Histograms showing the spheroid formation efficiency of Hep-12 cells infected with pri-miR-31 or control lentivirus. One hundred cells per well were plated (n=6). Spheroids (≥100 μm) were counted under a stereomicroscope. **(D)** The expression of miR-31 was analyzed in purified ɑ2δ1^+^ PLC/PRF/5 cells which were infected with pri-miR-31 or control lentivirus. Error bars indicate S.D. **(E)** Representative photographs demonstrating the spheroids formed by sorted ɑ2δ1^+^ PLC/PRF/5 cells which were infected with pri-miR-31 or control lentivirus. **(F)** Histograms showing the spheroid formation efficiency of sorted ɑ2δ1^+^ PLC/PRF/5 cells which were infected with pri-miR-31 or control lentivirus. One hundred cells per well were plated (n=6). Spheroids (≥100 μm) were counted under a stereomicroscope. **(G&H)** The tumor formation ability of Hep-12 cells stably infected with pri-miR-31 lentivirus was assayed in NOD/SCID mice by transplanted 1000 cells per site subcutaneously (n=5).

To validate if miR-31 could suppress the properties of ɑ2δ1^+^ HCC TICs, we overexpressed miR-31 in the TIC-enriched Hep-12 cell line and ɑ2δ1^+^ TICs sorted from the HCC PLC/PRF/5 cells by lentivirus infection. The transduction of the lentivirus harboring pri-miR-31 expression cassette resulted in remarkable elevation of miR-31 in both the Hep-12 cells and purified ɑ2δ1^+^ fraction from PLC/PRF/5 cell line as demonstrated by qRT-PCR (Figure 1A&1D). We then evaluated the effect of miR-31 on the *in vitro* self-renewal capability of ɑ2δ1^+^ TICs by spheroid formation assay. The spheroid formation efficiency decreased from 29.7% to 18.5% after overexpressing miR-31 in Hep-12 cells and decreased from 34.1% to 21.6% after overexpressing miR-31 in sorted ɑ2δ1^+^ subset form PLC/PRF/5 cell line (Figure 1B&1C, 1E&1F, P<0.05). We finally tested the tumor formation ability of the TIC-enriched Hep-12 cells after miR-31 overexpression. As shown in Figure 1G&1H, the tumor formation ability of Hep-12 cells was significantly suppressed when miR-31 was overexpressed. These results demonstrate that overexpression of miR-31 does inhibit the self-renewal and tumorigenic properties of ɑ2δ1^+^ HCC TICs.

### Knockdown of miR-31 enables HCC cells to acquire stem cell-like properties

**Figure 2:**
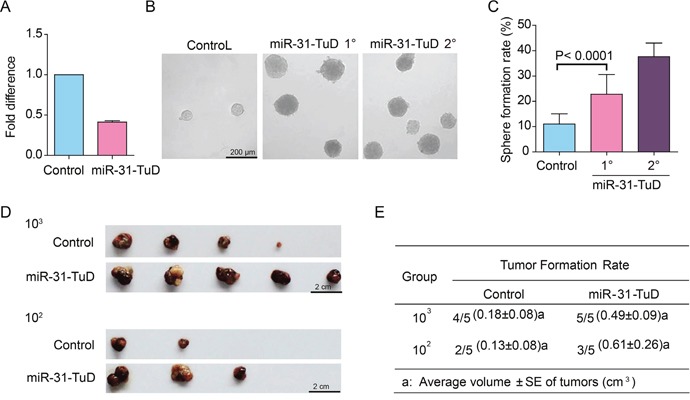
The effects of miR-31 knockdown on the stem cell-like properties of HCC cells **(A)** The fold change of miR-31 in PLC/PRF/5 cells upon infection with lentivirus harboring expression cassette of Tough Decoy (TuD) RNA against miR-31. Error bars indicate S.D. **(B)** Representative photographs showing the spheroids formed by PLC/PRF/5 cells with miR-31 knockdown. **(C)** Histograms showing the spheroid forming efficiency change of PLC/PRF/5 cells after miR-31 knockdown. The ability of the spheroids formed by PLC/PRF/5 cells with miR-31 knockdown to form secondary spheroid was also shown (miR-31-TuD 2°). One hundred cells per well were plated (n=6). Spheroids (≥100 μm) were counted under a stereomicroscope. **(D&E)** The tumorigenicity of PLC/PRF/5 cells infected with miR-31 TuD RNA or empty lentivirus (n=5). The tumor volumes are presented as average ± S.E.

To further address whether downregulation of miR-31 is sufficient to reprogram HCC cells into TIC-like cells, we knocked down the expression of miR-31 in PLC/PRF/5 cells using the tough decoy (TuD) RNA method [25]. The miR-31 level was downregulated by 59% after PLC/PRF/5 cells were infected with lentivirus harboring the Tough Decoy (TuD) RNA expression cassette against miR-31 (Figure 2A). We next carried out spheroid formation assay to measure if these cells could acquire *in vitro* self-renewal ability. As shown in Figure 2B&2C, the spheroid formation efficiency was remarkably promoted following knockdown of miR-31 in PLC/PRF/5 cells. Furthermore, these spheroids could be clonally expanded in subsequent serial propagation with increased efficiency when they were dissociated into single cells, demonstrating that the PLC/PRF/5 cells acquired *in vitro* self-renewal capability after miR-31 knockdown.

We also evaluated the tumorigenic potential of these PLC/PRF/5 cells with miR-31 knocked-down in NOD/SCID mice. The tumorigenic potential was enhanced remarkably when miR-31 was knocked down, as evidenced with higher tumor formation rate and larger tumor volume in the miR-31 knocked-down group than the control group (Figure 2D & 2E).

The above results attest that knockdown of miR-31 does reprogram HCC cells into TIC-like cells.

### MiR-31 negatively regulates the expression of stem cell-related genes

**Figure 3:**
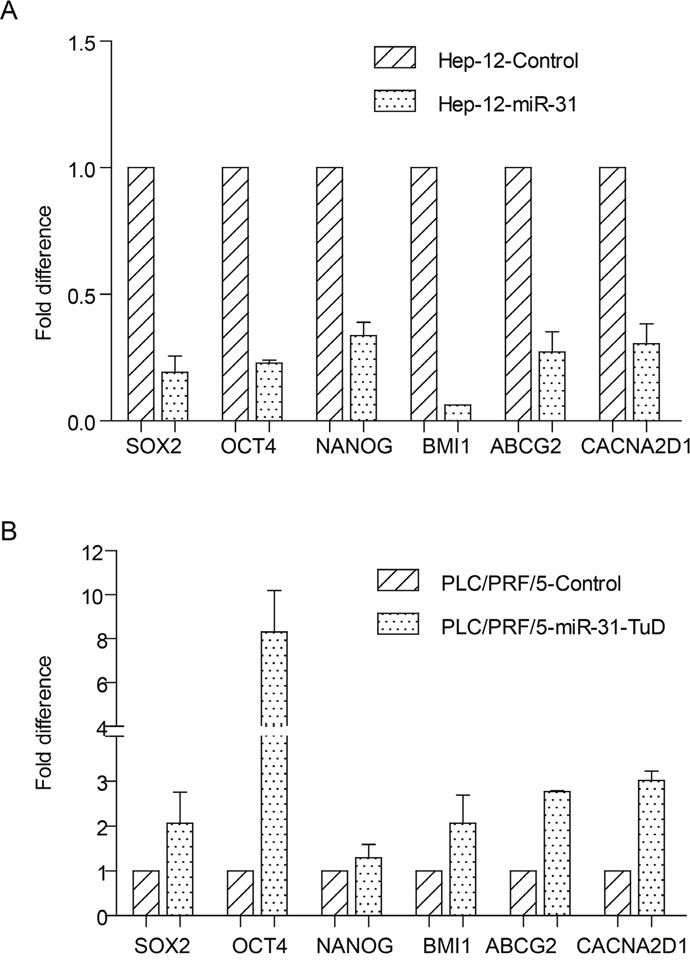
The effects of miR-31 on the expression of stem cell-related genes **(A&B)** The expression of indicated stem cell-related genes in the TIC-enriched Hep-12 cells overexpressing miR-31 **(A)** and in the PLC/PRF/5 cells with miR-31 knocked-down **(B)** was compared with respective vector alone controls using qRT-PCR. Error bars indicate S.D.

We next analyzed the effects of miR-31 on the expression of stem cell-related genes including *SOX2*, *OCT4*, *NANOG*, *BMI1*, *ABCG2* and *CACNA2D1* through real-time quantitative RT-PCR assay. The results showed that these genes were downregulated by about 66~94% after ectopic expression of miR-31 in the TIC-enriched Hep-12 cells, compared with vector alone control (Figure 3A). On the contrary, knockdown the expression of miR-31 in the PLC/PRF/5 cells led to the upregulation of these genes (Figure 3B). These data are in consistent with the suppressor role of miR-31 in α2δ1^+^ TIC properties.

### MiR-31 targets *ISL1* directly

To understand the underlying mechanisms involved in the suppression effects of miR-31 on α2δ1^+^ HCC TICs, we sought to identify the molecule(s) that miR-31 directly targeted. We have obtained a number of such candidate molecules by merging the list of upregulated genes in the α2δ1^+^ HCC TICs obtained by Affymetrix microarray mRNA hybridization (our unpublished data) with predicted target genes of miR-31 using the miRWalk algorithm. Of these candidate genes, we have selected *ISL1* for detailed study since our unpublished data indicate that this gene plays critical roles in the determination of α2δ1^+^ HCC TIC potential.

**Figure 4:**
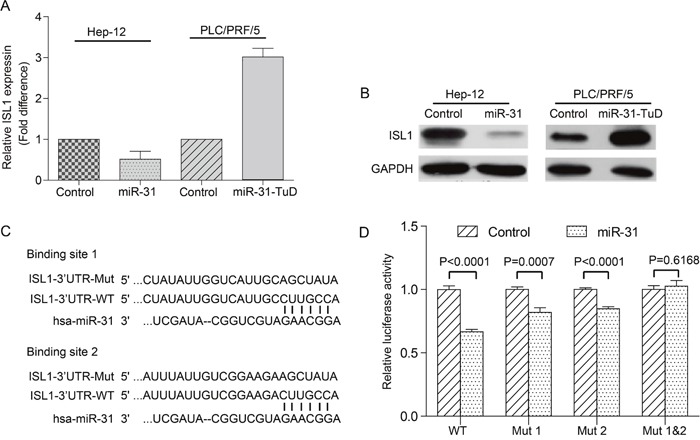
miR-31 targets *ISL1* directly **(A&B)** The expression of endogenous *ISL1* mRNA (A) and protein (B) in the TIC-enriched Hep-12 cells overexpressing miR-31 and in the PLC/PRF/5 cells with miR-31 knocked-down was compared with respective vector alone controls using qRT-PCR and Western blot, respectively. **(C)** Sequence alignment of human miR-31 seed sequence with the 3′-UTR of *ISL1*. The mutant sequences in the matched binding sites were also shown. **(D)** Luciferase reporter assay demonstrating the effects of miR-31 on *ISL1* reporter activity. The data are the mean±S.D. of three independent experiments.

We first validated if miR-31 could negatively regulate the expression of *ISL1*. As shown in Figure 4A&4B, ectopic expression of miR-31 in the TIC-enriched Hep-12 cells resulted in remarkably downregulation of *ISL1* at both mRNA and protein levels. On the other hand, knockdown miR-31 in PLC/PRF/5 cells led to significant upregulation of *ISL1* mRNA and protein.

We then performed luciferase reporter assay to test if the putative miR-31 binding site(s) in the 3′-UTR of *ISL1* is responsible for the repressor role of miR-31 on *ISL1*. We have identified two putative miR-31 binding sites in the 3′-UTR of *ISL1* through bioinformatics analysis (Figure 4C). The 3′-UTR of *ISL1* flanking the two putative binding sites was then cloned into the downstream of firefly luciferase, and reporter vectors with either the putative binding site 1, site 2, or both sites mutated were also made. Comparing with vector alone control, miR-31 could significantly inhibit the activity of luciferase with wild-type 3′-UTR of *ISL1* by as many as about 33% (Figure 4D, P<0.05). When either of the two putative miR-31 binding sites was mutated, the restraining effect of luciferase activity resulting from miR-31 overexpression reduced to a lesser degree (Figure 4D, P<0.05). Furthermore, the suppressive effect of miR-31 on luciferase reporter activity disappeared after both the putative miR-31 binding sites were mutated (Figure 4D, P>0.05). Hence, the two miR-31 binding sites in the 3′-UTR of *ISL1* are indeed responsible for the suppressive effect of miR-31 on the reporter activity, suggesting that miR-31 negatively and directly modulates the expression of *ISL1* through targeting its 3′-UTR.

### MiR-31 is downregulated in ɑ2δ1^+^ HCC TICs

**Figure 5:**
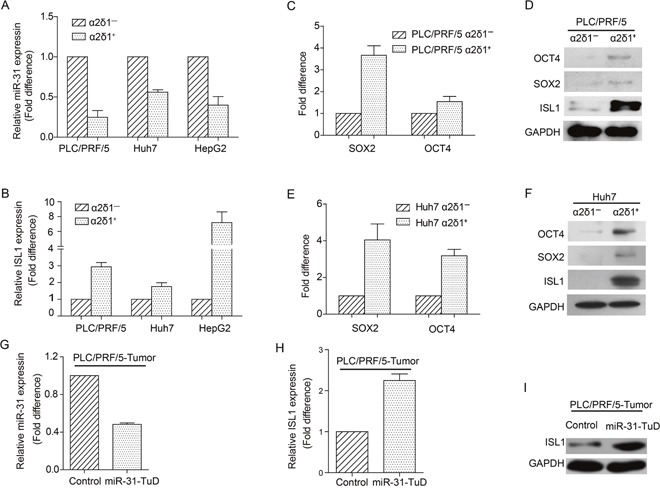
The expression of miR-31 and ISL1 in ɑ2δ1+ TICs **(A&B)** The expression levels of miR-31 (A) and *ISL1* mRNA (B) in ɑ2δ1^+^ TICs sorted from indicated cell lines were compared with respective ɑ2δ1^−^ subpopulations. Data presented as fold difference of ɑ2δ1^+^ TICs over respective negative counterparts, which were defined as 1 (calibrator). Error bars indicate S.D. **(C-F)** The expression of indicated stem cell-related genes and *ISL1* in both the ɑ2δ1^+^ TICs and their negative counterparts sorted from the cell lines PLC/PRF/5 **(C&D)** and Huh7 **(E&F)** was detected by qRT-PCR **(C&E)** and Western blot **(D&F)**, respectively. **(G)** qRT-PCR result showing the fold change of miR-31 level in the tumors formed by PLC/PRF/5 cells with miR-31 knockdown, compared with vector alone control cells, which was defined as 1 (calibrator). Error bars indicate S.D. **(H&I)** The expression of *ISL1* at both mRNA **(H)** and protein **(I)** levels in the tumors formed by both PLC/PRF/5 cells with miR-31 knockdown and vector alone control cells was detected by qRT-PCR and Western blot, respectively.

In view of the suppressive role of miR-31 on ɑ2δ1^+^ TICs by targeting *ISL1* directly, we measured the expression of miR-31 and *ISL1* in ɑ2δ1^+^ HCC TICs and ɑ2δ1^−^ non-TICs sorted from HCC cell lines Huh7 and PLC/PRF/5. As shown in Figure 5A, the expression of miR-31 was downregulated remarkably in ɑ2δ1^+^ HCC TICs compared with their negative counterparts, while the expression of its target gene *ISL1* was upregulated in ɑ2δ1^+^ HCC TICs compared with respective ɑ2δ1^−^ subsets (Figure 5B). Consistently, the expression of the stem cell-related genes such as *SOX2* and *OCT4* were also detected at higher levels in ɑ2δ1^+^ HCC TICs (Figure 5C-5F). Furthermore, the inverse correlation between miR-31 and ISL1 was also observed in the tumors formed by PLC/PRF/5 cells with miR-31 knocked-down. These data confirm that miR-31 is downregulated in ɑ2δ1^+^ TICs and is negatively correlated with the level of ISL1.

### Rescue expression of *ISL1* overcomes the TIC-suppression effect of miR-31

**Figure 6:**
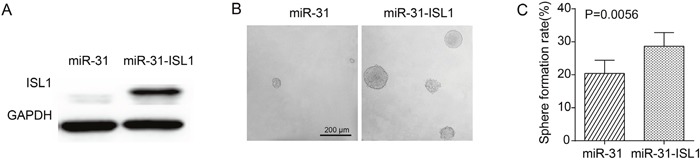
Rescue expression of ISL1 overcoming the TIC-suppression effect of miR-31 **(A)** Western blot result demonstrating the increased ectopic expression of *ISL1* after Hep-12 cells overexpressing miR-31 were infected with lentiviruses harboring the expression cassette without the 3′UTRs of *ISL1*. **(B)** Representative photographs showing the spheroids formed by Hep-12 cells overexpressing miR-31 and infected with lentiviruses harboring ISL1 expression cassette or control lentiviruses. **(C)** Histograms showing the spheroid formation efficiency of Hep-12 cells overexpressing miR-31 and infected with lentiviruses harboring ISL1 expression cassette or control lentiviruses. One hundred cells per well were plated (n=6). Spheroids (≥100 μm) were counted under a stereomicroscope.

To further validate if *ISL1* could functionally overcome the restraining effects of miR-31 on ɑ2δ1^+^ HCC TICs, rescue experiments were performed by introducing the expression cassette of ISL1 without 3′-UTR into miR-31 overexpressing Hep-12 cells. As shown in Figure 6, ectopic expression of *ISL1* in Hep-12 cells overexpressing miR-31 resulted in spheroid formation ability recovered from 20.4% to 28.7%, indicating that downregulation of *ISL1* is necessary for the suppression roles of miR-31 in HCC TICs. The data further confirm that *ISL1* is a *bona fide* target of miR-31.

### Epigenetic modification contributes to the downregulation of miR-31 in ɑ2δ1^+^ HCC TICs

**Figure 7:**
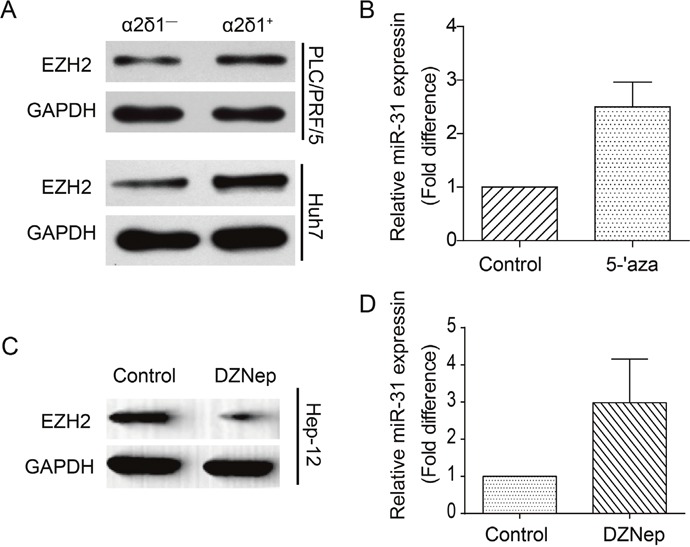
The effects of epigenetic modification on the expression of miR-31 in ɑ2δ1+ TICs **(A)** Western blot result demonstrating the expression of EZH2 in both ɑ2δ1^+^ and ɑ2δ1^−^ subpopulations sorted from indicated cell lines. GAPDH serves as loading control. **(B)** qRT-PCR analysis of the expression of miR-31 in the TIC-enriched Hep-12 cells after the treatment with 5-aza-dC, compared with the vehicle only control. Error bars indicate S.D. **(C)** Western blot result showing the expression of EZH2 in Hep-12 cells after the treatment with DZNep. **(D)** The fold change of miR-31 level in Hep-12 cells after the treatment with DZNep as detected by qRT-PCR. Error bars indicate S.D.

There are reports demonstrating that miR-31 repression in various kinds of tumors including HCC is attributed to epigenetic repression caused by DNA methylation and EZH2-mediated H3K27me3 epigenetic mark [18]. To address if the repression of miR-31 expression in ɑ2δ1^+^ HCC TICs is also caused by similar epigenetic mechanisms, we first checked if the histone methyltransferase EZH2 was highly expressed in ɑ2δ1^+^ HCC TICs. Western blot result indicated that EZH2 was indeed upregulated in ɑ2δ1^+^ HCC TICs compared with ɑ2δ1^−^ HCC non-TICs (Figure 7A). We then tested if DNA demethylation could enhance the expression of miR-31 by treatment Hep-12 cells with the DNMT inhibitor, 5-aza-2′-deoxycytidine (5-aza-dC). As shown in Figure 7B, the expression of miR-31 was upregulated about 2.5 times in Hep-12 cells treated with 2 μmol/L 5-aza-dC for 72h. Finally, we treated Hep-12 cells with EZH2 inhibitor 3-deazaneplanocin A (DZNep). The level of EZH2 was significantly decreased following the 48h treatment with 5 μmol/L DZNep as demonstrated by Western blot (Figure 7C), while the expression of miR-31 was upregulated following EZH2 inhibition (Figure 7D). These results indicate that DNA methylation and polycomb-mediated histone methylation both contribute to miR-31 silencing in ɑ2δ1^+^ HCC TICs.

## DISCUSSION

Numerous studies have identified many miRNAs that were aberrantly expressed in HCC TICs, and were demonstrated as key modulators of the stemness of HCC TICs by targeting various tumor-suppressive or oncogenic signaling pathways [12, 14, 26–29]. These studies have led us to a profound understanding of the characteristics of HCC TICs. In this study, we confirmed that miR-31 was significantly downregulated in the ɑ2δ1^+^ HCC TICs through epigenetic silencing, and played as a negative regulator of the stem cell-like properties of ɑ2δ1^+^ HCC TICs.

MiR-31 is among the most frequently altered miRNAs in human cancers. However, miR-31 may play a diametrically opposite role in different cancer types. For example, miR-31 is considered as a tumor suppressor in Ewing sarcoma, since it can significantly reduce cell proliferation due to increased apoptosis or increased length of G1-phase and reversely affect the invasiveness of Ewing sarcoma cell lines [21]. On the contrary, miR-31 acts as an oncogenic miRNA in human lung cancer tissues by targeting specific tumor suppressors LATS2 (large tumor suppressor kinase 2) and PPP2R2A (protein phosphatase 2, regulatory subunit B, alpha). Knockdown of miR-31 can substantially repress lung cancer cell growth and tumorigenicity [20]. Furthermore, miR-31 is found significantly downregulated in lung cancer side population (SP) cells, which are enriched for cancer stem cells, compared with non-SP cells. Repression of miR-31 can cause growth inhibition of lung cancer SP cell *in vitro* and notably can prevent SP cell differentiation [17], suggesting that the lower level of miR-31 in SP cells may benefit the maintenance of stem cells characteristics of SP cells. Here, we observed that miR-31 suppressed the stem cell properties of ɑ2δ1^+^ HCC TICs, further indicating that the roles of miRNA-31 on tumor cells are cell type-dependent.

It was previously reported that miR-31 could directly target PKC-ζ (epsilon), integrin alpha5 (ITGA5), or HDAC2 to exert its tumor suppressor roles [18, 19, 30]. Here, we identified that miR-31 targeted *ISL1*, possibly by degrading its mRNA, to suppress the self-renewal and tumorigenic properties of α2δ1^+^ HCC TICs. The gene *ISL1* encodes a member of the LIM/homeodomain family of transcriptional factors. The encoded protein ISL1 is central to the development of pancreatic cell lineages and may also be required for motor neuron generation and the correct development of striatonigral pathway in mouse [23, 31, 32]. More importantly, recent studies show that ISL1 marks pluripotent cardiovascular progenitor cells and is required for proliferation, survival, and migration of recently defined second heart field progenitors [24, 33]. It would be interesting to determine if ISL1 is essential for maintenance of α2δ1^+^ HCC TIC properties, and if so, what is the underlying mechanism(s)?

In summary, our study documents that downregulation of miR-31 may contribute to the self-renewal and tumorigenic properties of ɑ2δ1^+^ HCC TICs. Restoration the expression of miR-31 may represent a strategy to limit HCC TICs, and therefore may be used in conjunction with classical chemotherapy for the management of liver cancer. Further study is required to determine if the HCC stem cell marker ɑ2δ1 could regulate the expression of ISL1 or/and EZH2 through Ca^2+^ signaling. Nevertheless, these findings enhance our understanding the role of miRNA in the regulation of TICs, which will contribute to the elimination of TICs.

## MATERIALS AND METHODS

### Cell lines and cell culture

The establishment of HCC cell lines Hep-11 and Hep-12 were described in our earlier paper [22]. The HCC cell lines HepG2 and PLC/PRF/5 were originated from American Type Culture Collection (ATCC, Manassas, VA), and the Huh7 cell line was originated from Japan Society for the Promotion of Science (Tokyo, Japan). All cell lines were cultured in RPMI-1640 medium supplemented with 10% fetal bovine serum, 100 U/ml penicillin, and 100 mg/ml streptomycin (Invitrogen, Grand Island, NY, USA) in a humidified atmosphere of 5% CO_2_ at 37°C.

### RNA extraction and quantitative real time-PCR (qRT-PCR)

Total RNA was extracted from cells using miRNeasy Mini Kit according to the vendor's instruction (QIAGEN, Valencia, CA, USA). To quantify mature miRNAs, a polyA tail was appended to 200 ng total RNA by polyA polymerase (New England Biolabs, Beverly, MA, USA), followed by reverse transcription with an Oligo-d(T)_15_ adapter primer and Moloney murine leukaemia virus reverse transcriptase (MMLV, Invitrogen). For mRNA detection, cDNAs were synthesized from 3 μg total RNA using an oligo-d (T)_15_ primer and MMLV. Sequences of all primers were listed in Supplementary Table 1. The cDNA products from miRNA or mRNA were then used as templates in qRT-PCR by using SYBR Green PCR Master Mix (Toyobo, Osaka, Japan) on Applied Biosystems 7500 real time PCR system. The relative amount of miRNAs or mRNA was normalized to *U6* or *GAPDH*, respectively, which was used as internal control. Fold change of target miRNAs or mRNA expression was calculated by the 2^-DDCt^method where DC_t_=C_t_ (target)-C_t_ (reference).

### Protein extraction and western blotting analysis

Protein was extracted from cells by using Radio-Immunoprecipitation Assay buffer (Suolaibo Biotechnology Co. Ltd, Shanghai, China) containing complete protease and phosphatase inhibitor cocktail (Roche, Mannheim, Germany), and was then separated through 10% sodium dodecyl sulfate polyacrylamide gel, transferred onto PVDF membrane (Millipore, Billerica, CA), probed with a rabbit polyclonal antibody against ISL1 protein (1:8000 dilution; Abcam, Cambridge, UK), or a rabbit monoclonal antibody against EZH2 (1:1000 dilution; Epitomics, Burlingame, CA), or a mouse polyclonal antibody against GAPDH (1:10^5^ dilution, Roche), followed by incubation with HRP-conjugated goat anti-rabbit or anti-mouse IgG (Jackson ImmunoResearch Laboratories, West Grove, PA) secondary antibody. Signals were visualized by using the method of chemiluminescence (Millipore).

### Plasmid construction, cell transfection and stable cell line establishment

The human pri-miR-31 sequence containing miR-31 pre-miRNA and its flanking sequences on both sides was amplified from genomic DNA using the primers listed in Supplementary Table 1, and was subsequently cloned into pcDNA3.0 vector and lentivirus shuttle vector plenti6 (Invitrogen). For knockdown of miR-31 expression, tough decoy (TuD) RNA against miR-31 (5′-CGggatccGACGGCGCTAGGATCATC AACAGCTATGCCAGATCTCATCTTGCCTCAAGTATTCTGGTCACAGAATACAACAGCTATGCCAGATCTCATCTTGCCTCAAGATGATCCTAGCGCCGTCTTTTTTctcgagCGG-3′) were designed according to reference [25]. The TuD sequence was cloned into lentiviral shuttle vector plenti6-U6 vector.

For miR-31 sensor vector, the 3′-UTR of *ISL1* gene including potential miR-31 binding sites was amplified from cDNAs prepared from Hep-12 cells, and the corresponding mutant 3′-UTRs were obtained by overlap-extension PCR. These amplification products were subsequently cloned into the downstream of firefly luciferase gene of the pGL3-control plasmid (Promega, Madison, WI).

Lentiviral constructs were transfected with the ViraPower Packaging Mix (Invitrogen) into 293FT cells to generate lentivirus. Cells infected with virus are selected by 5 μg/ml blasticidin (Invitrogen). The pool of antibiotic-resistance cells were used for subsequent assay.

### Flow cytometry

Cells were dissociated with 0.02% EDTA/PBS, then resuspended into 1×PBS buffer and stained with either fluorescence-conjugated specific antibody 1B50-1 which specifically recognizes the HCC stem cell marker ɑ2δ1 isoform 5 or isotype-matched mouse IgG3 at 4°C for 30 min. The antibody 1B50-1 was directly labeled with Fluorescein isothiocyanate using the Lightning-Link™ Fluorescein kit, according to the vendor's protocol (Innova Biosciences Ltd., Cambridge, UK). Labeled samples were sorted using a FACSAria II ™ flow cytometer (BD Biosciences, San Jose, CA).

### Spheroid formation assay

To detect spheroid formation efficiency, dissociated cells were plated in Ultra-Low attachment 96-well plates at 100 cells per well (Corning Incorporated Life Sciences, Acton, MA), and were cultured in Dulbecco's modified Eagle's medium/F12 (Invitrogen) supplemented with 50 ng/ml basic fibroblast growth factor, 10 ng/ml HGF, 50 ng/ml epidermal growth factor (Peprotech, Rocky Hill, NJ), 1% methylcellulose (Sigma-Aldrich, St Louis, MO) and B27 (Invitrogen)) in a humidified atmosphere of 5% CO_2_ at 37°C.. After 2–3 weeks, spheres were counted under a stereomicroscope (Olympus, Tokyo, Japan).

### Tumorigenicity assay in NOD/SCID mice

To assay the tumor formation efficiency, cells at indicated numbers were suspended in 100 μl of a 1:1 mix of plain RPMI 1640 medium and Matrigel (BD Biosciences, Bedford, M A, USA) and transplanted subcutaneously into the armpit of 4- to 6- week-old female Non-obese diabetic/severe combined immunodeficient (NOD/SCID) mice (NOD.CB17-prkdcscid/NcrCrl, Vitalriver, Beijing, China). Tumor formation was monitored weekly. 7-8 weeks later, mice were sacrificed and the tumors were dissected. The tumor volume was determined using the formula V = L×W^2^×0.5, where L and W represent the largest and the smallest diameters, respectively.

All the animal experiments were performed in accordance with the guidelines of the use of laboratory animals and approved by the Animal Care and Use Committee of Peking University Cancer Hospital.

### Dual-luciferase reporter assay

PLC/PRF/5 cells grown in 24-well plates were co-transfected with 300 ng pGL3 reporter construct, 500 ng pcDNA3.0 or pcDNA3.0-miR-31 and 26 ng pRL-TK expressing renilla luciferase using Lipofectamine™ 2000 (Invitrogen). After 48h, cell lysates were made using 1×passive lysis buffer (Promega Corporation) according to the manufacturer's instructions. Firefly and Renilla luciferase activities were measured by a FLUOstar Optima illuminometer (BMG Labtech, Offenburg, Germany) using the dual-luciferase reporter assay kit (Promega). Firefly luciferase activity was normalized to that of renilla luciferase for each sample.

### Statistical analysis

GraphPad Prism 6 software was used to analyze the data. Differences between each group were assessed by t-test. A p value of less than 0.05 was considered statistically significant.

## SUPPLEMENTARY MATERIALS TABLE


