# Supplementary material for: MicroRNA-31 suppresses the self-renewal capability of α2δ1+ liver tumor-initiating cells by targeting ISL1
Source: Oncotarget. 2017 Sep 21;8(50):87647–57. doi: 10.18632/oncotarget.21140 (PMC5675660; doi:10.18632/oncotarget.21140)
Supplement: Supplementary file 1 [file oncotarget-08-87647-s001.pdf]

# MicroRNA-31 suppresses the self-renewal capability of α2δ1⁺ liver tumor-initiating cells by targeting *ISL1*

## SUPPLEMENTARY MATERIALS

### Supplementary Table 1: Primer sequences

| **qRT-PCR primers** | | |
|---|---|---|
| **Gene Name** | **Sense (5’------3’)** | **Anti-sense (5’------3’)** |
| *miR-31* | AGGCAAGATGCTGGCATAGCT | GCGAGCACAGAATTAATACGAC |
| *SOX2* | ACATGAACGGCTGGAGCAAC | AGGAAGAGGTAACCACAGGG |
| *Oct4* | GACAACAATGAAAATCTTCAGGAGA | CTGGCGCCGGTTACAGAACCA |
| *NANOG* | TGCCTCACACGGAGACTGTC | TGCTATTCTTCGGCCAGTTG |
| *BMI1* | AGCAGCAATGACTGTGATGC | CAGTCTCAGGTATCAACCAG |
| *ABCG2* | GGAGGCCTTGGGATACTTTGAA | GAGCTATAGAGGCCTGGGGATTAC |
| *CACNA2D1* | ACAGCAAGTGGAGTCAATCA | ACTGCTGCGTGCTGATAAGA |
| *ISL1* | TCCCTATGTGTTGGTTGC | ACTCCGCACATTTCAAAC |
| *U6* | CGCTTCGGCAGCACATATAC | TTCACGAATTTGCGTGTCAT |
| *GAPDH* | GACCCCTTCATTGACCTCAAC | CTTCTCCATGGTGGTGAAGA |
| **Primers for Gene Cloning** | | |
| **Gene Name** | **Sense (5’------3’)** | **Anti-sense (5’------3’)** |
| *miR-31* | CGGGATCCACCTCCTGTGCCTAACTAC | CCGCTCGAGAGGTGTGTCCAAGGAATAG |
| *ISL1-3’-UTR* | TCGACCGGTCGTAGAGGATTTATATTCAAGG | CCAATGCATGACATATAATTCAAAGACCAC |
| *ISL1-3’-UTR-1bind-Mutation* | CATTGCAGCTATAAACAGGAGCTCCAGCAAAAGCGC | CCTGTTTATAGCTGCAATGACCAATATAGAAACAGC |
| *ISL1-3’-UTR-2bind-Mutation* | GAAGAAGCTATACTTTTCATGTCATTTGACATTTTTTG | AAGTATAGCTTCTTCCGACAATAAATAATAAATTAC |
